# Apparent Motion Can Impair and Enhance Target Visibility: The Role of Shape in Predicting and Postdicting Object Continuity

**DOI:** 10.3389/fpsyg.2013.00035

**Published:** 2013-02-01

**Authors:** Peter J. Lenkic, James T. Enns

**Affiliations:** ^1^Department of Psychology, University of British ColumbiaVancouver, BC, Canada

**Keywords:** visual masking, apparent motion, shape perception, prediction, postdiction

## Abstract

Some previous studies have reported that the visibility of a target in the path of an apparent motion sequence is impaired; other studies have reported that it is facilitated. Here we test whether the relation of *shape* similarity between the inducing and target stimuli has an influence on visibility. Reasoning from a theoretical framework in which there are both predictive and postdictive influences on shape perception, we report experiments involving three-frame apparent motion sequences. In these experiments, we systematically varied the congruence between target shapes and contextual shapes (preceding and following). Experiment 1 established the baseline visibility of the target, when it was presented in isolation and when it was preceded or followed by a single contextual shape. This set the stage for Experiment 2, where the shape congruence between the target and both contextual shapes was varied orthogonally. The results showed a remarkable degree of synergy between predictive and postdictive influences, allowing a backward-masked shape that was almost invisible when presented in isolation to be discriminated with a *d*′ of 2 when either of the contextual shapes are congruent. In Experiment 3 participants performed a shape-feature detection task with the same stimuli, with the results indicating that the predictive and postdictive effects were now absent. This finding confirms that shape congruence effects on visibility are specific to shape perception and are not due to either general alerting effects for objects in the path of a motion signal nor to low-level perceptual filling-in.

## Introduction

When two stimuli are presented in close spatio-temporal proximity we experience a single object in motion. Although such *apparent motion* is experienced without effort by the viewer, it is only achieved after a number of complex problems have been solved. These include problems of image correspondence (Ramachandran and Anstis, 1986), the relative spatial position of elements (Nijhawan, 1994; Eagleman and Sejnowski, 2000; Krekelberg and Lappe, 2000), and visual masking of one stimulus by the other (Breitmeyer and Ogmen, 2000, 2006; Enns and Di Lollo, 2000). One might reasonably predict from these challenges that a stimulus in motion would be seen less accurately than a static stimulus of similar duration and size. In the present paper, we demonstrate that visibility can sometimes be impaired and at other times enhanced by the relations between stimuli making up the perceptual object in an apparent motion sequence.

### Evidence for prediction and postdiction in perception

The role of prediction is emphasized in recent theories of spatio-temporal processing (Nijhawan, 1994; Enns and Lleras, 2008; Mathewson et al., 2010; Roach et al., 2011). As one example of a study of motion predictability on target visibility, Schwiedrzik et al. (2007) presented a target within various phases of the up-and-down motion path of a secondary stimulus and reported that target visibility was especially reduced when the target coincided with the middle portion of the motion path. In contrast, visibility was increased for targets at the end-points of the path, and when there was only a single preceding motion stimulus or a single following motion stimulus. Schwiedrzik et al. (2007) referred to this impairment as “motion masking,” in keeping with the earlier use of this term by Yantis and Nakama (1998). Similar results have also been been reported by Hidaka et al. (2011, 2012), Khuu et al. (2010), and Souto and Johnston (2012).

In another study, Roach et al. (2011) presented pairs of inducer stimuli to the left and right of central fixation, oscillating up-and-down over several cycles. A target Gabor patch was presented in the path of one of these inducers, and its timing adjusted so that it appeared either at the end of the motion sequence or the beginning. The target was also presented either in or out of spatial phase with the inducer. The participant’s task was to report whether the target appeared to the left or right of fixation. The results indicated that target visibility was lowest when the inducing stimuli moved away from the target location and it was highest when it was predictable from both the temporal and spatial phase of the inducer. Thus, contrary to Schwiedrzik et al. (2007), motion predictability was a *benefit* to target visibility in this task, not an impairment.

Prediction, or forward-going expectations, are only part of what occurs in a motion sequence. Postdiction, or a revisionist history of what has just occurred, also influences the visibility of a target in motion (Di Lollo et al., 2000; Eagleman and Sejnowski, 2000; Lleras and Moore, 2003; see also Kolers and Pomerantz, 1971; Kolers and von Grunau, 1976). The theoretical mechanism for these influences is often referred to as *object updating*, because the visual system seems to give a revisionist interpretation specifically to perceptual objects, not to the image as a whole (see review by Enns et al., 2009). That is, there is a powerful bias to interpret changes to a scene as the consequence of a single object in motion, rather than as the sudden appearance of unexpected new objects, or as the consequence of a moving background in the context of a stationary single object. This bias offers heuristic benefits to a visual system faced with chaotic input, but at the same time it incurs a cost in certain conditions. The cost is that target features seen at point A in time may be overwritten and rendered less visible, or even invisible, by the target features presented at point B. This is the main idea behind what has come to be called *object substitution* masking (e.g., Di Lollo et al., 2000; Lleras and Moore, 2003; Moore and Lleras, 2005; Enns, 2008).

### The role of shape

At what level of representation are the predictive and postdictive mechanisms at work when interpreting an object in motion? Extant theories of how motion relates to target visibility have been described as falling into three camps (Souto and Johnston, 2012). In one camp are researchers who give their participants a detection task (i.e., reporting whether a stimulus is present or absent along a motion path), thereby emphasizing image-level processes. For example, Hidaka et al. (2011) showed that motion path predictability lead to a decrement in target detection, and they conclude that motion masking is the result of an early visual interaction between a physical stimulus (the target) and an illusory percept (the interpolated motion path between stimulus inducers). Souto and Johnston (2012) expanded on this idea, reporting that motion masking depended on the targets and inducers sharing the same isoluminant colors. In a second camp, researchers have demonstrated that object-level competition between inducers and target also plays a role in motion masking (Yantis and Nakama, 1998; Liu et al., 2004). These authors demonstrate that more than detection-level processes are involved by giving their participants shape-discrimination tasks. In a third camp, Schwiedrzik et al. (2007) and Roach et al. (2011) go a step further, by arguing that when masking is attenuated by motion path consistency, it demonstrates the role of predictive processes at play, over, and above an object-level competition between stimuli.

Although Schwiedrzik et al. (2007) and Roach et al. (2011) show that predictable targets can attenuate masking (i.e., reduce the visibility impairment caused by motion), they do not examine the role of shape consistency between stimuli and inducers, focusing only on spatio-temporal consistency. To be fair, Schwiedrzik et al. (2007) discuss the possibility that the shape dissimilarity between the stimuli in motion and the target may have played a role in the impairments that they and Yantis and Nakama (1998) reported. This way of thinking also raises the possibility that the predictive benefits of Roach et al. (2011) may have occurred because of the greater similarity between inducing and target shapes in their study.

Here we focus on the role of *shape continuity* in the visibility of a target in an apparent motion sequence. Specifically, we compare the influences that arise from forward-acting (predictive) processes with those that derive from backward-acting (postdictive) processes (see also Hogendoorn et al., 2008). If we find that both processes are at work, we can then ask questions about their relative magnitude and whether they combine in an additive way (indicating independent processes) or interactively (pointing to synergistic processes).

It may also be important to distinguish between previous studies in which the target stimulus was unrelated to the motion inducing stimulus (e.g., Yantis and Nakama, 1998; Khuu et al., 2010), offering greater opportunity for masking, versus those in which the target stimulus was a component of the motion inducing stimulus (e.g., Hidaka et al., 2011). As such, we begin with a study in which the target to be perceived is itself part of the motion sequence.

To address these questions, we designed a target discrimination task in which the effects of a preceding shape and a following shape could be evaluated, first independently (Experiment 1), and then jointly (Experiment 2). We did this by varying the motion congruence between the central target shape and the contextual shapes (preceding, following). To anticipate the results, we report strong predictive and postdictive influences on target visibility, along with a great deal of synergy between these influences.

In a final experiment (Experiment 3) we replicated the essential stimulus conditions of Experiment 2, but asked participants to perform a shape-feature *detection* task (presence versus absence) rather than a shape-*discrimination* task. This serves as an important control for the idea that predictive and postdictive processes specific to *shape* perception are influencing target visibility, as opposed to more primitive alerting process or image-level processes that boost the gain of all signals in the path. If the processes we are studying are shape specific, we anticipate that continuity in apparent motion will not have the same effect on a target detection task. And again, to anticipate the results, that is what we find.

## Experiment 1: Baseline Visibility

**Figure 1:**
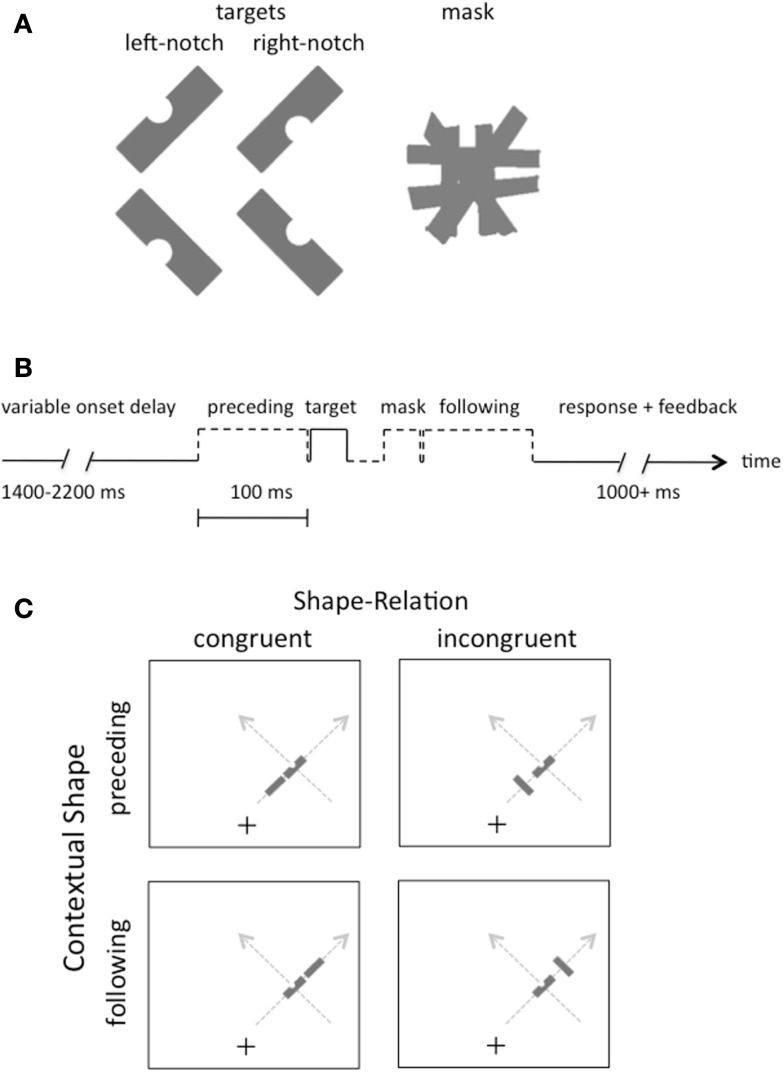
**(A)** Illustration of the four possible target shapes and the pattern mask in the experiments. Participants reported whether the target had a notch on the left or the right side, regardless of its slant. **(B)** Illustration of the sequence of events on each trial. **(C)** Illustration of the displays in Experiment 1. Gray arrows indicate the two possible motion directions on the right side of the screen; equivalent paths were possible on the left side (not shown).

To set the stage for a study of target visibility in the context of a three-frame motion sequence, we first compared the visibility of a target shape in isolation, with the visibility of a target either preceded or followed by a single shape. The spatial layout and temporal sequence is illustrated in Figure 1. We also varied the orientation of the preceding and following shapes, so that they were congruent or incongruent with the target. Three additional factors were varied to increase the generality of the findings and to minimize the possibility of strategic factors influencing the results. First, to ensure that target visibility would be measured at more than one level, we varied whether or not a pattern mask was presented immediately after the target and in the same spatial position (Breitmeyer and Ogmen, 2006). Second, we varied the spatial proximity between neighboring shapes at two levels, as this is often a critical factor in target visibility (Breitmeyer and Ogmen, 2006). Finally, the shapes were presented randomly to the right or left of fixation, and motion sequences were also either to the left or the right, so that observers were unable to predict where the shapes would appear and in what context (Enns and Di Lollo, 1997, 2000).

Participants were asked to report the location of a notch in each target shape, which could be either on the right or left side. Note that this task is immune from any decision-based biases arising from the orientation of the preceding or following shapes, or from the relation between these shapes and the target (congruent versus incongruent), since the only shape with a notch was the target, and the notch was equally often on the right or the left of this shape, independent of all other factors.

### Method

#### Participants

Fifteen university students participated in a 1-h session for extra-course credit or a $10 payment. All participants had normal or corrected-to-normal vision and were treated according to APA ethical guidelines as administered by the University of British Columbia.

#### Stimuli and apparatus

Rectangular gray shapes (gray level = 62%) were presented on an LCD monitor with a refresh rate of 60 Hz. The shapes subtended 2.5° × 1° of visual angle, were slanted either 45° or 135° from vertical (i.e., they had a positive or negative slant, see Figure 1A), and were presented on a white background. The pattern masks consisted of six rectangular shapes, as illustrated in Figure 1A, each oriented to differ slightly from the cardinal directions of vertical, horizontal, and oblique. This pattern subtended 2.5° × 2.5° of visual angle. The target shape had a semicircular notch on one side. A fixation cross was centered horizontally on the screen, but positioned 5.5° below the vertical center, so that the shapes were presented above fixation.

The contextual shape that preceded or followed the target shape on some trials was identical to the target in size and luminance, but it did not have a notch, and it was spatially separated by a center-to-center distance of either 2.5° (near proximity condition) or 6.5° (far). The target was always presented 10.5° from the fixation point, but randomly to the left or right, with a positive or negative slant and with a notch randomly removed from its left or right side. The orientation of the preceding and following shapes was either congruent or incongruent with a linear motion trajectory.

The temporal sequence of events is illustrated in Figure 1B, with the target shape and preceding or following shape (when either was present) appearing 100 ms apart (stimulus onset asynchrony). The target had a duration of 33 ms, as did the mask, when present, and the target and mask were separated by an interval of 33 ms.

#### Procedure

Participants were seated with their eyes 57 cm from the display screen. They were instructed to maintain gaze on the cross in the bottom of the screen, using their peripheral vision to view the shapes. They were introduced to the task with 10 practice trials with much longer display durations and received feedback on each trial (the words “correct” or “incorrect” appeared at fixation), and the experimenter monitored this feedback during the practice trials and provided further verbal instruction when necessary to ensure they understood the task.

Each trial began with a variable onset interval (1400–2200 ms, in 200 ms steps) that began after the participant’s previous response. Participants registered their responses with one of two keys (“w” or “o”) and visual feedback consisting of a green or red colored text message at fixation indicated whether their response was “correct” or “incorrect,” respectively. Trials were presented in a random order, with equal representation of the three conditions (alone, preceding, and following) × 2 notch locations × 2 target orientations × 2 mask conditions. Among the preceding and following conditions, trials were further divided among congruent and incongruent shape relations and close and far proximity conditions. Participants completed a total of 768 trials, divided into eight blocks of 96 trials, with self-paced breaks between blocks.

#### Data analyses

In order to convert responses into hits and false-alarm rates that are amenable to a signal detection analysis, the proportion of left responses to left-notched targets were counted as hits and the proportion of right responses to left-notched targets were counted as false-alarms, for each participant. These rates were then used to calculate *d*′, a measure of sensitivity unaffected by response bias. Because proportions of 0 or 1 cause *d*′ to take on a value of infinity, hit, or false-alarm rates with these values were replaced with values of 0.01 and 0.99, respectively (MacMillan and Creelman, 1991), which placed a ceiling on *d*′ of 4.46.

### Results

**Figure 2:**
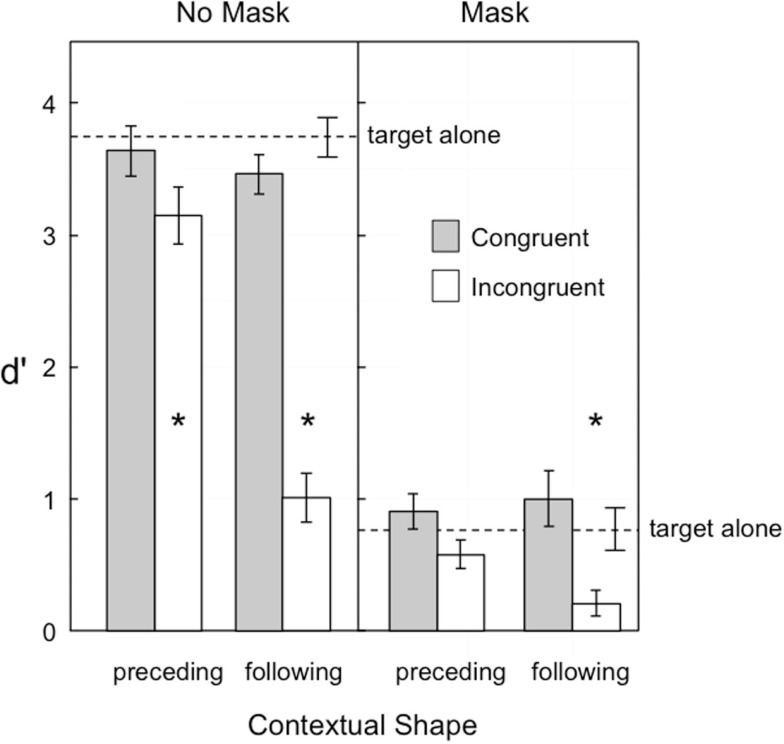
**Visibility of the target in Experiment 1, as indexed by *d*′**. Error bars represent ±1 SEM. The asterisks indicate those conditions in which target visibility was significantly reduced relative to the target alone condition.

Figure 2 shows target visibility in Experiment 1. Masking was clearly effective in reducing overall visibility, as the mean *d*′ was 3 with no mask and less than 1 with the mask. Shape congruency also played a large role in target visibility: congruent shape sequences resulted in larger *d*′ values than incongruent sequences at both levels of masking. The temporal order of the contextual shape also played a large role, with a preceding shape having less of an influence on target visibility than a following shape. Most important, the influence of shape congruence on visibility was greater for following shapes than preceding shapes, with an incongruent-following shape reducing visibility in the no-mask condition (*d*′ = 1.01) near the baseline level in the masking condition (*d*′ = 0.79), and in the mask condition reducing visibility to a *d*′ of near zero (*d*′ = 0.21). Contextual shapes that were near in proximity to the target generally led to lower levels of visibility (*d*′ = 1.64) than contextual shapes that were farther away (*d*′ = 1.85). These observations were supported by the following statistical analyses.

A repeated measures ANOVA examined the factors of temporal order (2) × congruency (2) × masking (2) × proximity (2). All main effects were significant: temporal order [*F*_(1,14)_ = 19.17, *p* = 0.00063], congruence [*F*_(1,14)_ = 105.26], mask [*F*_(1,14)_ = 369.07], and proximity [*F*_(1,14)_ = 6.52, *p* = 0.023], as were the two-way interactions of temporal order × congruence [*F*_(1,14)_ = 65.40], temporal order × proximity [*F*_(1,14)_ = 9.50 *p* = 0.0081], temporal order × mask [*F*_(1,14)_ = 17.28, *p* = 0.00097], mask × congruence [*F*_(1,14)_ = 59.57], and mask × proximity [*F*_(1,14)_ = 5.03, *p* = 0.042]. The only significant three-way interactions were temporal order × congruence × mask [*F*_(1,14)_ = 18.09, *p* = 0.00080] and congruence × mask × proximity [*F*_(1,14)_ = 5.37, *p* = 0.036]. All other effects were not significant (*p*s > 0.094).

Simple effect tests on the critical temporal order × congruence interaction indicated that, although the congruency effect was much greater in the following than preceding condition, congruent shapes were nonetheless more visible than incongruent shapes in both conditions: [*F*_(1,14)_ = 234.70] and [*F*_(1,14)_ = 15.08, *p* = 0.0017], respectively.

Additional comparisons tested whether target visibility in the preceding and following shape conditions was improved or impaired relative to the target presented alone. The asterisks in Figure 2 indicate which of these comparisons were significant, based on a Bonferroni-adjusted family wise alpha of *p* < 0.05. With no mask, only the two incongruent conditions resulted in significant reductions in visibility, preceding [*F*_(1,14)_ = 16.53, *p* = 0.0012] and following [*F*_(1,14)_ = 190.86, *p* < 0.0001]. When the mask was present only the following incongruent condition showed a significant visibility reduction [*F*_(1,14)_ = 21.15, *p* = 0.0004].

### Discussion

These results establish an important baseline for us to explore how prediction and postdiction combine in their influence when a target is seen in the context of a larger motion sequence. In summary, the results show that shape congruence in a motion sequence plays a critical role in influencing the visibility of a target shape, such that when the shapes are congruent, visibility is similar to when the same target is presented briefly in isolation. However, when the shapes are incongruent there is a serious reduction in visibility, with this reduction being much greater for an incongruent shape that follows the target (postdiction based on the incongruent shape impairs visibility) than for an incongruent shape that precedes it (prediction based on an incongruent shape has little consequence).

These results are broadly consistent with previous reports of motion masking (Yantis and Nakama, 1998; Schwiedrzik et al., 2007; Hogendoorn et al., 2008), in that placing a target in a motion sequence can be detrimental to its visibility under some conditions (e.g., when following shapes are incongruent). These results are also consistent with previous reports that backward masking of shape is generally more detrimental to visibility than forward masking (Breitmeyer and Ogmen, 2006). Finally, they are consistent with object updating theory (Enns et al., 2009), which proposes that human vision is biased to process a spatio-temporal sequence of stimuli as the same object translating in space-time. To the extent that this bias is supported by a spatio-temporally consistent motion display (here the congruent condition), the visibility of a target shape in an apparent motion sequence is not impaired.

## Experiment 2: Visibility in an Apparent Motion Sequence

In this experiment we measured the visibility of a target shape in a three-frame apparent motion sequence, while varying whether the preceding and following shapes were congruent or incongruent with the overall motion trajectory. By comparing these data with those in Experiment 1, we were able to gage the extent to which congruency in the two contextual shapes made additive or synergistic contributions to target visibility.

### Method

**Figure 3:**
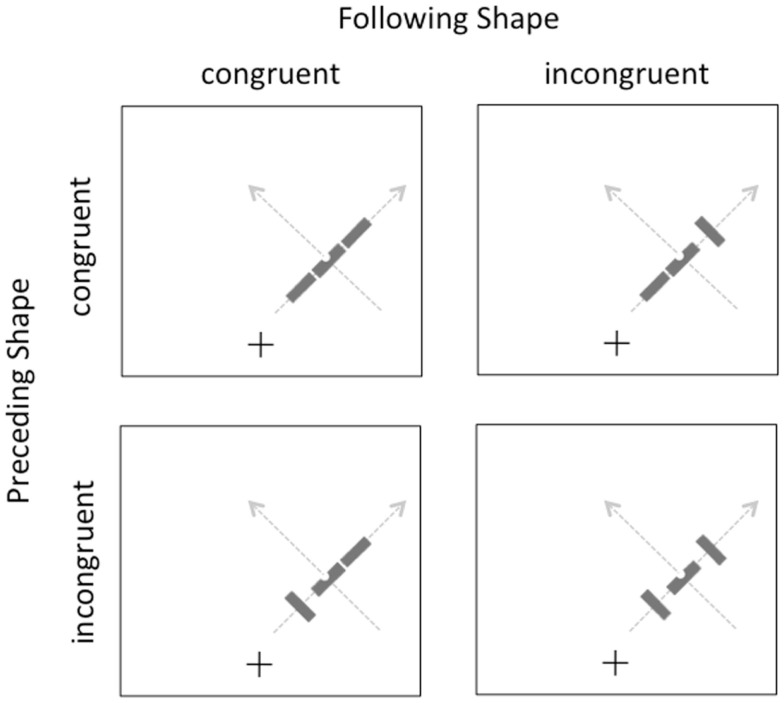
**Illustration of the displays in Experiment 2**. Gray arrows indicate the two possible motion directions on the right side of the screen; equivalent paths were possible on the left side (not shown).

The methods were identical in this experiment to the previous one, with the exception that the participants were 15 different university students and all of the displays now had both preceding and following contextual shapes in addition to the target. These shapes could be independently congruent or incongruent with overall motion trajectory, as illustrated in Figure 3. The target was always congruent with the overall motion trajectory. Participants again completed a total of 768 trials, divided into eight blocks of 96 trials, with self-paced breaks between blocks.

### Results

**Figure 4:**
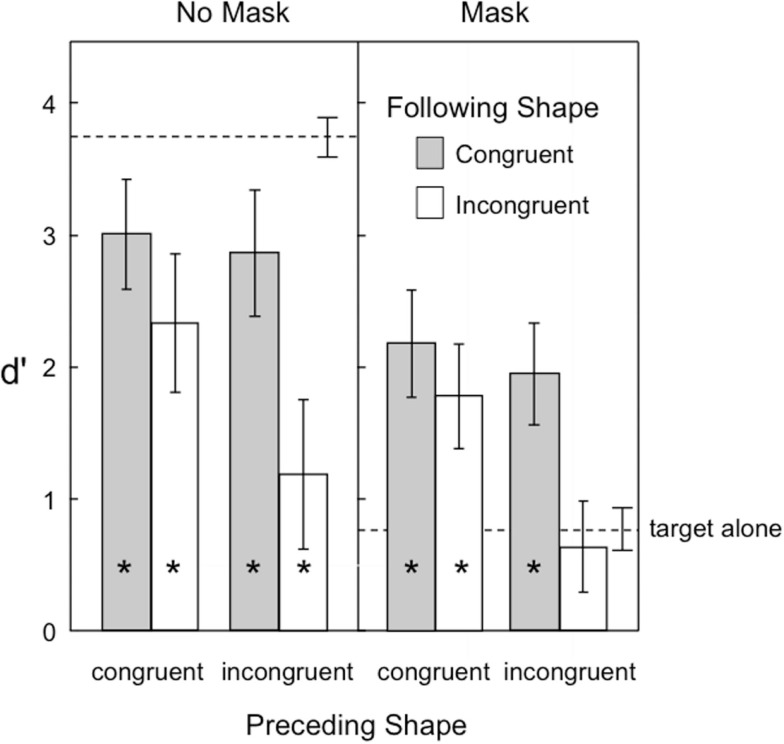
**Visibility of the target in Experiment 2, as indexed by *d*′ in a shape-discrimination task**. Error bars represent ±1 SEM. The asterisks indicate those conditions in which target visibility was significantly reduced or increased relative to the target alone condition.

Figure 4 shows the target visibility in Experiment 2. As in the previous experiment, backward masking was effective in reducing overall visibility of the target. Shape congruence also provided a significant benefit to target visibility. One important new finding was observed in the backward masking condition (right panel of Figure 4). Here the target shapes in the three-frame motion sequence were now even *more visible* than when the same target shape was presented in isolation.

A second important finding was that the effects of preceding and following shapes were synergistic. Specifically, congruent contextual shapes preceding or following target shapes were both beneficial to target visibility, but the consequences of sandwiching the target shape between two incongruent shapes was catastrophic to its visibility. Even without a backward pattern mask to reduce visibility (left panel in Figure 4), two incongruent context shapes reduced target visibility to levels similar to that of a solitary target followed by a pattern mask. In the masking condition (right panel), two incongruent context shapes again reduced visibility to that same low level.

A third finding was that the detrimental effects of backward pattern masking on target visibility were largely overcome by placing the target into a three-frame sequence of apparent motion. In contrast to the baseline influence of backward masking, which was about 3 *d*′ units when a target was presented in isolation (compare target alone visibility for no masking versus masking in Figure 4), backward masking was less than a 1 *d*′ unit effect when either the preceding or following shape was congruent in a motion sequence (compare target visibility for congruent shapes in the no masking versus masking conditions in Figure 4).

Finally, as in Experiment 1, contextual shapes that were near in proximity to the target generally led to lower levels of visibility (*d*′ = 1.91) than contextual shapes that were farther away (*d*′ = 2.06). These observations were supported by the following statistical analyses.

A four-way repeated measures ANOVA was conducted with the following factors: 2 preceding shape congruence × 2 following shape congruence × 2 mask × 2 proximity conditions. Target visibility was higher when the preceding shape was congruent than when it was incongruent [*F*_(1,14)_ = 32.28, *p* = 0.000057], and it was higher when the following shape was congruent than when it was incongruent [*F*_(1,14)_ = 40.19, *p* = 0.00018]. Backward masking reduced target visibility [*F*_(1,14)_ = 92.73], and close proximity was marginally significant in reducing target visibility [*F*_(1,14)_ = 3.96, *p* = 0.066]. The two-way interaction of preceding shape congruence × following shape congruence was significant [*F*_(1,14)_ = 27.97, *p* = 0.00011], as was the four-way interaction of all factors combined [*F*_(1,14)_ = 5.87, *p* = 0.030]. Bonferroni tests (family wise alpha = 0.05) of the interaction indicated that target visibility in all four congruency conditions was lower than the single target baseline when there was no mask. However, when there was a backward pattern mask, target visibility in three of the four congruency conditions was now significantly *greater* than the single target baseline. Only when the target was placed between two incongruent shapes was target visibility not improved over that of a single target.

A comparison of the effects of backward pattern masking on the single target condition (Experiment 1) with the motion sequence conditions (Experiment 2) indicated that backward masking was more detrimental to single target visibility than it was to each of the four motion conditions formed by combining preceding congruence with following congruence, in the order shown in Figure 4 [*t*_(28)_ = 10.96, *t*_(28)_ = 11.34, *t*_(28)_ = 9.40, and *t*_(28)_ = 11.91].

### Discussion

These results indicate that an apparent motion sequence has both detrimental and beneficial effects on the visibility of a target shape embedded in the sequence. In comparison to a target shape presented briefly in isolation, placing the target in the center of a three-frame motion sequence reduces its visibility somewhat (less than 1 *d*′ unit). However, this reduction is greater when the following contextual shape is incongruent with the motion trajectory implied by all three shapes, and it is even greater when both contextual shapes are incongruent with this trajectory. This latter finding is consistent with Yantis and Yakama’s (1998) previous reports of motion masking, in which they found significant reductions in letter visibility within the motion path of two circle stimuli, which were highly dissimilar in shape since the circles contained only curved edges whereas the letters consisted solely of straight lines.

The truly novel result of this study is the benefit that occurs for target visibility in the context of backward pattern masking. Here the results show that in comparison to a target shape presented briefly in isolation and then masked, placing the target in the center of a three-frame motion sequence increases its visibility quite significantly (more than 1 *d*′ unit). This finding runs counter to some previous reports of motion masking (Yantis and Nakama, 1998; Schwiedrzik et al., 2007; Hogendoorn et al., 2008). However, this finding is consistent with theories based on the constructs of prediction and postdiction in motion processing, including the RECOD model (Breitmeyer and Ogmen, 2000) and object updating theory (Enns et al., 2009). Consistent with these theories, when a target shape is embedded within a motion path that allows for prediction and postdiction based on shape, a target shape can become more visible than it would otherwise be.

What are we to make of the finding that motion contributed to an enhancement of target visibility in the masking condition, but not in the no-masking condition? One possibility is that this reflected a ceiling effect. If so, then participants were already discriminating shapes at a near optimal level in the no-mask solitary target conditions, with no room for improvement. As such, the enhancement in visibility deriving from a shape-consistent motion trajectory was measurable until overall visibility had been degraded with a backward pattern mask.

Another possibility is that the visibility benefit (relative to a single target) only occurs under backward masking conditions because the shape-based predictions allow for the recovery of features in the target that have become suppressed by the backward mask. On this account, reentrant processes of object substitution make it difficult to access the original target features that have been substituted by the mask features (Di Lollo et al., 2000). The benefit of the congruent motion sequence is that this substitution process no longer occurs within the context of predictive motion. Indeed, one of the ways these mechanisms could play an active role in such a visibility benefit is through what Otto et al. (2006) refer to as “grouping-based feature inheritance.” That is, because the target is perceived to be the same object as the inducers, merely at a different spatial-temporal location, the target feature (i.e., the notch) that would otherwise be backward-masked may actually be seen by participants as belonging to the following shape, which is not masked. Such feature migrations or inheritance effects have been documented in many previous studies of masking (Wilson and Johnson, 1985; Enns, 2002; Otto et al., 2006).

## Experiment 3: Shape Congruency Does Not Influence Target Detection

This experiment tested whether the influences of apparent motion on target shape visibility were specific to shape perception, or whether they applied to the mere detection of a stimulus. One reason for posing this question is because of mixed previous results in the motion masking literature. For example, although Kolers (1963) failed to find evidence of motion masking using a detection task, others reported motion masking effects using detection, identification, and discrimination tasks (Yantis and Nakama, 1998; Schwiedrzik et al., 2007; Hogendoorn et al., 2008; Hidaka et al., 2011). Moreover, Gellatly and colleagues (Gellatly et al., 2006; Pilling and Gellatly, 2009) and Hogendoorn et al. (2008) have both reported significant interactions of task and masking, with masking being much more effective on shape discrimination than on shape detection. These findings strongly hint that it is not only the detection of a shape’s presence that is influenced by the motion trajectory, but rather it is the determination of the target’s detailed shape characteristics that are affected.

### Method

The methods were identical to Experiment 2, with the exception that the participants were 15 different university students, the target shapes now had a notch on a random one-half of the trials, and only one proximity condition was tested (the far condition). The participant’s task was to report whether the target shape had a notch (target present) or not (target absent). Participants again completed a total of 768 trials, divided into six blocks of 128 trials. The data were analyzed by counting correct reports of a notch as hits and counting reports of a notch on target absent trials as a false alarm. *d*′ Values were then calculated as in the previous experiments.

### Results

**Figure 5:**
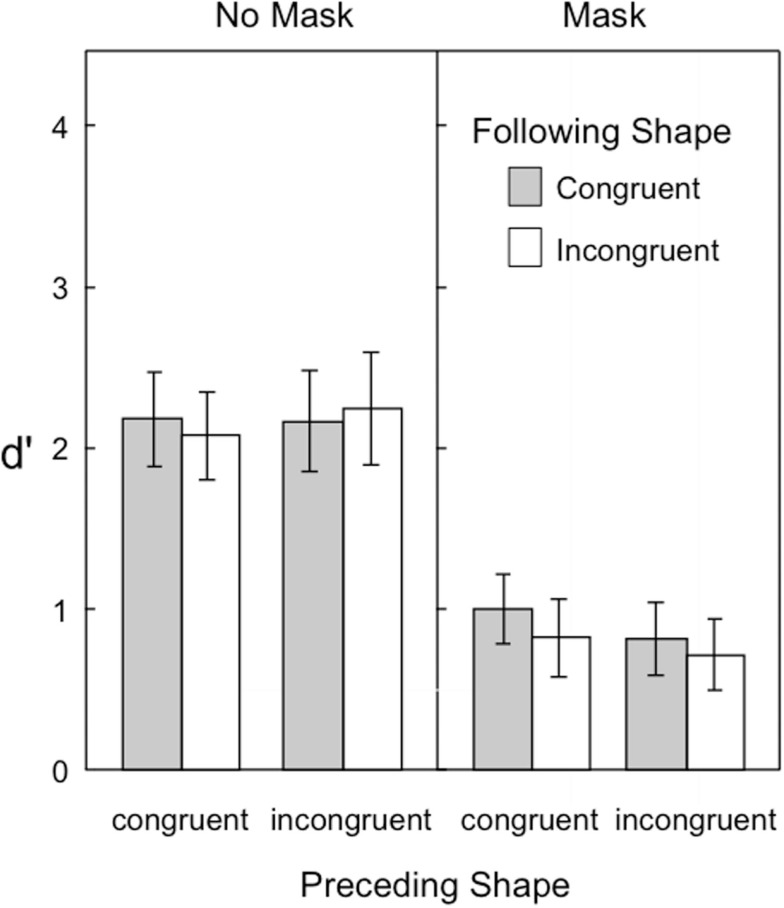
**Visibility of the target in Experiment 3, as indexed by *d*′ in a shape detection task**. Error bars represent ±1 SEM.

Figure 5 shows the target visibility in Experiment 3. As in previous experiments, the backward pattern mask was effective in reducing the overall visibility of the target. Yet, unlike the discrimination task (Experiments 1 and 2), the congruency of the preceding and following shapes had no measurable influence on the detection task. Another noticeable difference between experiments was the reduction in *d*′ in the no-mask condition. A comparison of Figures 4 and 5 shows that target visibility as measured by the detection task is considerably reduced overall from that of the shape-discrimination task. These observations were supported by the following statistical analyses.

A three-way repeated measures ANOVA was conducted with the following factors: 2 preceding shape congruence × 2 following shape congruence × 2 mask conditions. The backward pattern mask reduced target visibility [*F*_(1,14)_ = 149.49]. The only other significant effect was the interaction of mask × preceding shape congruence [*F*_(1,14)_ = 6.47, *p* = 0.023; all other *p*s > 0.085]. Simple main effect follow-ups revealed that there was an effect of preceding shape congruence when the mask was present [*F*_(1,14)_ = 5.79, *p* = 0.030] but not when the mask was absent [*F*_(1,14)_ = 1.41, *p* = 0.25]. This suggests that a congruent preceding shape is able to help to detect a target which is followed by a backward mask, but the congruence of the preceding shape makes no difference in detecting an unmasked target.

A comparison of these results with Experiment 2 was conducted with a mixed ANOVA involving the between-groups factor of two tasks (discrimination, detection) and the within-subjects factors of two preceding shape congruence and two following shape congruence. Target visibility differed marginally according to task [*F*_(1,28)_ = 4.19, *p* = 0.050], with the detection task showing lower target sensitivity than the discrimination task. Also, target visibility differed significantly according to preceding and following shape congruence [*F*_(1,28)_ = 30.03, and *F*_(1,28)_ = 40.00]; however, this effect was moderated by two-way interactions of task × preceding congruence [*F*_(1,28)_ = 24.02], task × following congruence [*F*_(1,28)_ = 29.02], and preceding × following congruence [*F*_(1,28)_ = 11.06, *p* = 0.0025]. Finally, the three-way interaction of task × preceding × following congruence was significant [*F*_(1,28)_ = 20.00, *p* = 0.00012]. This three-way interaction follows from the finding that the two-way preceding × following shape congruence interaction was significant for the discrimination task in Experiment 2, but not significant for the detection task of experiment 3.

We also conducted analyses examining the effect of masking across the different tasks. For this, we used a 2 × 2 ANOVA with mask as a within-groups factor and task (discrimination, detection) as a between-groups factor. This ANOVA showed a significant effect of mask [*F*_(1,28)_ = 197.42], and significant interaction of task × mask. Follow-up simple main effect analyses revealed that the masking effect was significant for both the discrimination and detection tasks, but larger in the latter than the former [*d*′ difference = 0.60, *F*_(1,28)_ = 39.93; *d*′ difference = 1.28, *F*_(1,28)_ = 181.77].

### Discussion

These results indicate that the shape congruence effect on motion masking in Experiments 1 and 2 is specific to the task of discriminating target shapes. It does not apply to merely detecting the presence or absence of a target feature in the motion sequence. While this is generally consistent with the report from Gellatly et al. (2006) that detection tasks are influenced less by backward masking than discrimination tasks, it also extends this finding to the consequences of contextual shapes in a motion sequence. That is, Experiments 2 and 3 taken together, show that contextual shape congruency has a strong influence on target visibility when the task is to discriminate among two possible shape possibilities, but that it has no influence when the task is merely to detect the presence of the shape’s distinctive feature.

## General Discussion

In this study we examined how the perception of a target’s shape is influenced by its relation to the shapes that precede and follow it in an apparent motion sequence. In a first experiment, we established the baseline visibility of a target shape, both when it was presented in isolation and when it was preceded or followed by a single shape. The results showed a reduction in visibility when either the preceding or following shapes were incongruent, though this visibility impairment was greater when the incongruent shape was following rather than preceding. This finding is consistent with what many previous reports that it is more effective to mask a target shape with a neighboring shape that follows rather than precedes the target (Enns and Di Lollo, 2000; Breitmeyer and Ogmen, 2006).

In a second experiment we studied the combined effects of preceding and following shapes. The novel result here was a considerable *benefit* for target visibility from a congruent three-frame motion sequence. The results indicated that in comparison to an isolated target shape, presented briefly, and backward masked, a target in the center of a three-frame motion sequence was increased in its visibility by more than 1 *d*′ unit. This finding runs counter to some previous reports of motion masking (Yantis and Nakama, 1998; Schwiedrzik et al., 2007; Hogendoorn et al., 2008; Khuu et al., 2010; Hidaka et al., 2011), but is consistent with theories that appeal to the constructs of prediction and postdiction (Breitmeyer and Ogmen, 2006; Enns et al., 2009). Moreover, the present finding offers a resolution to the mixed results of previous research, which did not systematically study the role of *shape congruence* in motion masking phenomena. In contrast to those mixed results, the present findings suggest that *motion masking* (a visibility impairment) is most likely to occur when target and contextual shapes are different, and motion enhancement (a visibility benefit) is most likely to occur when target and contextual shapes are the same. This is because the contextual shapes influence target visibility through expectations (both predictive and postdictive) that are based on the available evidence about *shape* (Breitmeyer and Ogmen, 2000; Enns et al., 2009).

In the third experiment, when the participant’s task was to merely detect the presence or absence of the target feature, without having to indicate its precise location, all shape congruency effects disappeared. This finding helps to confirm that the visibility effects measured in Experiments 1 and 2 were specific to binding shape features to precise locations in space, and were not reflecting more general mechanisms of arousal or alerting (Bachmann, 1984) nor of low-level perceptual filling-in (Hidaka et al., 2011; Souto and Johnston, 2012). Taken together, these results show that contextual shape congruency has a strong influence on target visibility when the task is to discriminate among two possible shape possibilities, but it has no influence when the task is merely to detect a target feature. This confirms that the prediction and postdiction processes evoked by the contextual shapes in these motion sequences were concerned with *shape*.

The results of this study also provide (1) a comparison of the relative magnitude of predictive and postdictive effects on shape perception and (2) an analysis of whether these effects were additive or interactive. With regard to the first question, the results from both Experiments 1 and 2 indicate that postdiction has a stronger influence than prediction. This is seen in the greater impairments associated with an incongruent-following shape than an incongruent preceding shape, both when there was only one of these shapes (Figure 2) and when both contextual shapes were considered in combination (Figure 4). From the perspective of object updating theory (Enns et al., 2009), this asymmetry is a consequence of the way vision handles the task of keeping track of an object in motion. That is, the default interpretation that a sudden scene change is indicative of an object in motion biases the system to look for confirmatory evidence that the same shape features are now present in a new location. At the same time, unless attention has previously been focused on the specific features of the object, rather than simply its rough location, it will take some time to establish the appropriate links between the various features of the object and their locations in space. If during that time, the features have changed, the system may only have access to the target features currently on view. This leads to *object substitution* masking, which in the present study is expressed as target visibility that is especially reduced when the following shape is not a match for its shape features. As such, this is a consequence of our time-limited nervous systems, which is destined, by virtue of its slow processing speed, to be living “slightly in the past” (Eagleman and Sejnowski, 2000).

With regard to the second question, the data in Experiment 2 clearly point to an interactive (synergistic) pattern of influence for prediction and postdiction. That is, the combined *impairment* of having both preceding and following shapes be incongruent with the target was greater than could be predicted when only one of these shapes was incongruent on its own.

Importantly, this interaction was not a by-product of ceiling or floor effects on the accuracy measure, since the interaction occurred at two quite different levels of baseline visibility (compare the no mask and mask conditions in Figure 4). Such synergy is indicative of a single dynamic system, rather than of separate or dissociable mechanisms that combine their influences in a linear fashion.

Synergistic predictive and postdictive behavioral effects are also consistent with the neural feedback or recurrent neural activity that inspired theories of object updating (Breitmeyer and Ogmen, 2000; Moore et al., 2007; Enns et al., 2009). These theories are premised on conscious visual perception being the end product of a system containing neural projections that not only ascend the anatomical hierarchy, that is from regions of lower to higher-levels of representational complexity, but with neural connections that are horizontal (between regions with different specialization), and backward or reentrant to lower-level regions (Bullier et al., 1988; Felleman and Van Essen, 1991; Zeki, 1993). The conscious perception of a stimulus in these accounts is the result of the system reaching a stable state of resonance between the feedforward and reentrant signals. Recent evidence in support of this view comes from electrophysiological data from monkey (Fahrenfort et al., 2007) and from transcranial magnetic stimulation in humans (Ro et al., 2003; Hirose et al., 2005, 2007). For instance, Hirose et al. (2005, 2007) applied brief high-intensity magnetic pulses to the brain region MT/MT+ in human participants and reported that it disrupted masking and led to increased visibility of a target that would otherwise have been invisible. Notably for the present study, reentrant neural activity projecting from the MT cortex is also involved in motion perception, and thus may be the neural mechanism by which perception of a target in motion is influenced by signals generated by the contextual shapes surrounding a target shape (Liu et al., 2004; Muckli et al., 2005; Sterzer et al., 2006).

## Conflict of Interest Statement

The authors declare that the research was conducted in the absence of any commercial or financial relationships that could be construed as a potential conflict of interest.
